# Supplementary material for: A Comprehensive Evaluation of Taxonomic Classifiers in Marine Vertebrate eDNA Studies
Source: Mol Ecol Resour. 2025 Apr 17;25(7):e14107. doi: 10.1111/1755-0998.14107 (PMC12415807; doi:10.1111/1755-0998.14107)
Supplement: Supplementary file 1 — Data S1. [file MEN-25-e14107-s001.docx]

# Supplementary Figures


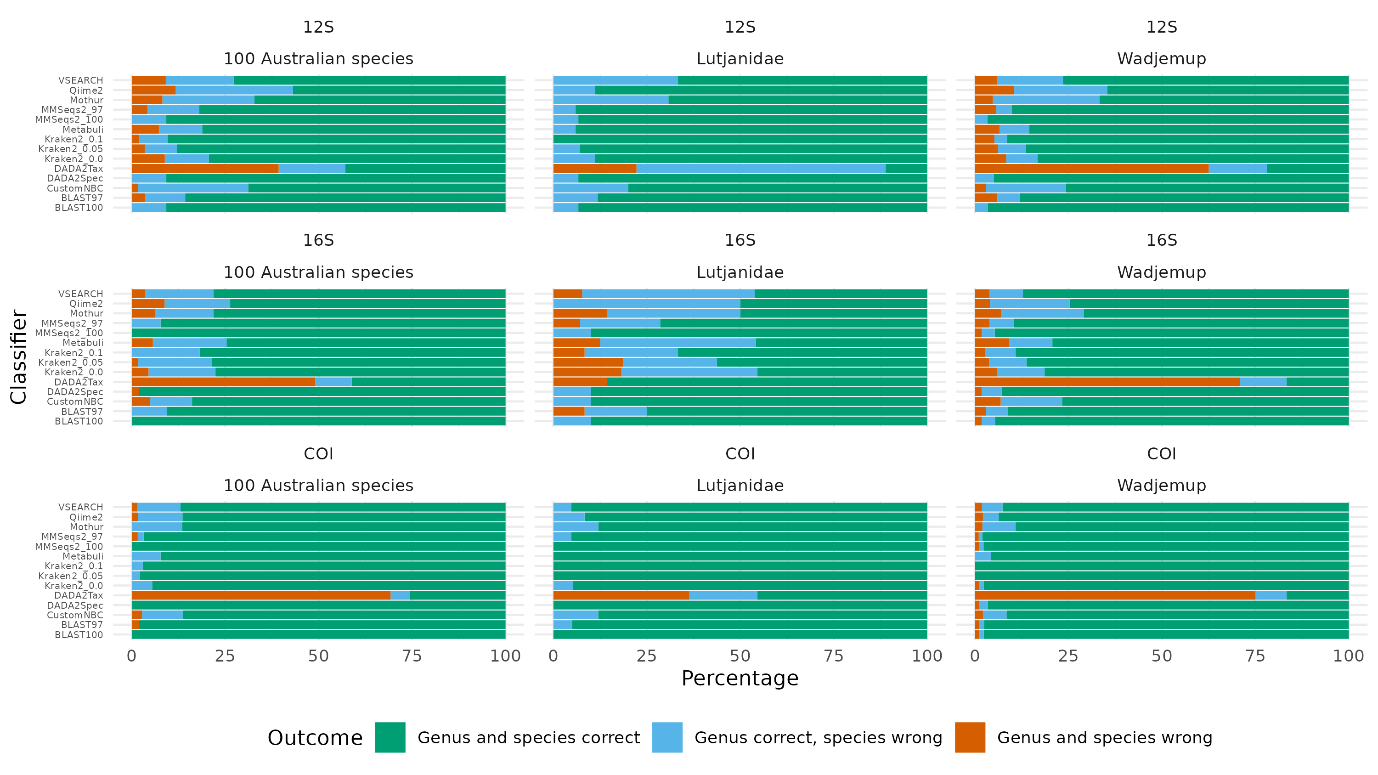


Figure S1: Error types across all databases and classifiers. Percentage of outcomes when genus and species predictions are both correct (green), when genus prediction is correct, but species prediction is incorrect (blue), and when genus and species predictions are both incorrect (orange).

# Supplementary Notes

## Supplementary Note 1: Full Entrez search terms for 12S, 16S, and CO1

The given taxonomy IDs can be extracted by using family names given in Table S1 and the NCBI Taxonomy API.

### 12S

esearch -db nuccore -query "

(txid29146[ORGN] OR txid107764[ORGN] …OR txid33574[ORGN]) AND (Small subunit [Title] OR 12S[Title] OR 12S ribosomal RNA[Title] OR 12S rRNA[Title]) NOT environmental sample[Title] NOT environmental samples[Title] NOT environmental[Title] NOT uncultured[Title] NOT unclassified[Title] NOT unidentified[Title] NOT unverified[Title] "

| efetch -format fasta > 12s_fish_nuccore.fasta

esearch -db gene -query "

(txid29146[ORGN] OR txid107764[ORGN] …OR txid33574[ORGN]) AND (Small subunit [Title] OR 12S[Title])"

| efetch -format docsum | xtract -pattern GenomicInfoType -element ChrAccVer ChrStart ChrStop | while IFS=$'\t' read acn str stp; do efetch -db nuccore -format fasta -id "$acn" -chr_start "$str" -chr_stop "$stp"; done | sed "s/>/>12S_/" > 12s_fish_gene.fasta

### 16S

esearch -db nuccore -query "

(txid29146[ORGN] OR txid107764[ORGN] OR txid54907[ORGN] … OR txid33574[ORGN]) AND (Small subunit [Title] OR 16S[Title] OR 16S ribosomal RNA[Title] OR 16S rRNA[Title]) AND (mitochondrion[Filter] OR plastid[Filter]) NOT environmental sample[Title] NOT environmental samples[Title] NOT environmental[Title] NOT uncultured[Title] NOT unclassified[Title] NOT unidentified[Title] NOT unverified[Title] "

| efetch -format fasta > 16S_fish_nuccore.fasta

esearch -db gene -query "

(txid29146[ORGN] OR … OR txid33574[ORGN]) AND (Small subunit [Title] OR 16S[Title])"

| efetch -format docsum | xtract -pattern GenomicInfoType -element ChrAccVer ChrStart ChrStop | while IFS=$’\t’ read can str stp; do efetch -db nuccore -format fasta -“d "$”cn" -chr_sta“t "$”tr" -chr_st“p "$”tp"; done | s“d "s/>/>16”_/" > 16S_fish_gene.fasta

### CO1

esearch -db nuccore -query '

(…. OR txid33574[ORGN]) AND ("cytochrome c oxidase 1"[Title] OR "cytochrome oxidase subunit I"[Title] OR COI[Title] OR COXI[Title] OR COX1[Title] OR "COX 1"[Title] OR "COX I"[Title] OR CO1[Title] OR C01[Title] OR "cytochrome oxidase I"[Title] OR "cytochrome oxidase subunit I"[Title] OR "cytochrome oxidase subunit 1"[Title] OR "cytochrome oxidase 1"[Title] OR "cytochrome c oxidase subunit I"[Title] OR "cytochrome c oxidase subunit 1"[Title])'

| efetch -format fasta > c01_fish_nuccore.fasta

esearch -db gene -query "

(txid29146[ORGN] OR txid107764[ORGN] OR txid54907[ORGN] OR txid134615[ORGN] OR txid7850[ORGN] OR txid1489899[ORGN] OR txid205120[ORGN] OR txid84619[ORGN] OR txid1072510[ORGN] OR txid7934[ORGN] OR txid8279[ORGN] OR txid83881[ORGN] OR txid303732[ORGN] OR txid170194[ORGN] OR txid117867[ORGN] OR txid31017[ORGN] OR txid316123[ORGN] OR txid163127[ORGN] OR txid143304[ORGN] OR txid69128[ORGN] OR txid81361[ORGN] OR txid150446[ORGN] OR txid31024[ORGN] OR txid490307[ORGN] OR txid88658[ORGN] OR txid463601[ORGN] OR txid1489799[ORGN] OR txid8065[ORGN] OR txid94935[ORGN] OR txid88661[ORGN] OR txid56718[ORGN] OR txid8253[ORGN] OR txid36203[ORGN] OR txid215350[ORGN] OR txid30757[ORGN] OR txid181401[ORGN] OR txid30850[ORGN] OR txid270592[ORGN] OR txid30908[ORGN] OR txid7865[ORGN] OR txid8157[ORGN] OR txid94930[ORGN] OR txid7805[ORGN] OR txid7802[ORGN] OR txid181420[ORGN] OR txid206122[ORGN] OR txid163122[ORGN] OR txid270595[ORGN] OR txid206106[ORGN] OR txid88666[ORGN] OR txid30828[ORGN] OR txid270610[ORGN] OR txid29142[ORGN] OR txid181415[ORGN] OR txid206140[ORGN] OR … OR txid33574[ORGN]) AND ("cytochrome c oxidase 1"[Title] OR "cytochrome oxidase subunit I"[Title] OR COI[Title] OR COXI[Title] OR COX1[Title] OR "COX 1"[Title] OR "COX I"[Title] OR CO1[Title] OR C01[Title] OR "cytochrome oxidase I"[Title] OR "cytochrome oxidase subunit I"[Title] OR "cytochrome oxidase subunit 1"[Title] OR "cytochrome oxidase 1"[Title] OR "cytochrome c oxidase subunit I"[Title] OR "cytochrome c oxidase subunit 1") NOT environmental sample[Title] NOT environmental samples[Title] NOT environmental[Title] NOT uncultured[Title] NOT unclassified[Title] NOT unidentified[Title] NOT unverified[Title] NOT "cytochrome b"[Title] NOT chromosome[Title] NOT "cytochrome P450"[Title] "

| efetch -format docsum | xtract -pattern GenomicInfoType -element ChrAccVer ChrStart ChrStop | while IFS=$'\t' read acn str stp; do efetch -db nuccore -format fasta -id "$acn" -chr_start "$str" -chr_stop "$stp"; done | sed "s/>/>16S_/" > c01_fish_gene.fasta
